# Supplementary material for: Increased transmissibility of SARS-CoV-2 lineage B.1.1.7 by age and viral load
Source: Nat Commun. 2021 Dec 13;12:7251. doi: 10.1038/s41467-021-27202-x (PMC8669007; doi:10.1038/s41467-021-27202-x)
Supplement: Supplementary file 1 — Supplementary Information [file 41467_2021_27202_MOESM1_ESM.pdf]

Supplementary Information

Increased Transmissibility of SARS-CoV-2 Lineage B.1.1.7 by Age and Viral Load

Frederik Plesner Lyngse[1,2,3,*],

Kåre Mølbak[3,4],

Robert Leo Skov[3],

Lasse Engbo Christiansen[5],

Laust Hvas Mortensen[6,7],

Mads Albertsen[8],

Camilla Holten Møller[3],

Tyra Grove Krause[3],

Morten Rasmussen[3],

Thomas Yssing Michaelsen[8],

Marianne Voldstedlund[3],

Jannik Fonager[3],

Nina Steenhard[3],

The Danish Covid-19 Genome Consortium,

Carsten Thure Kirkeby[4]

[*] Correspondence to Frederik Plesner Lyngse, fpl@econ.ku.dk. Affiliations: [1]Department of Economics & Center for Economic Behaviour and Inequality, University of Copenhagen; [2]Danish Ministry of Health; [3]Statens Serum Institut; [4]Department of Veterinary and Animal Sciences, Faculty of Health and Medical Sciences, University of Copenhagen; [5]DTU Compute; [6]Statistics Denmark; [7]Department of Public Health, University of Copenhagen; [8]Department of Chemistry and Bioscience, Aalborg University.

Supplementary Note 1: Descriptive Statistics

From December 21, 2020 (week 52) to February 21, 2021 (week 7), Denmark had 68,169 SARS-CoV-2 cases identified with RT-PCR, of which, 35,684 (52%) were selected for WGS and 28,383 (42%) came back with a genome (Table S1).

Table S1: Summary statistics for all positive cases

Week Number	First Weekday	Cases N	Selected for WGS N	%	With Genome N	%	With B.1.17 N	%
52	December 21, 2020	16,934	4,501	27	3,606	21	64	2
53	December 28, 2020	14,536	4,883	34	3,908	27	80	2
1	January 4, 2021	11,289	5,390	48	4,142	37	154	4
2	January 11, 2021	6,988	4,919	70	3,958	57	282	7
3	January 18, 2021	5,318	4,489	84	3,680	69	470	13
4	January 25, 2021	3,613	3,407	94	2,658	74	518	19
5	February 1, 2021	3,117	2,851	91	2,235	72	663	30
6	February 8, 2021	2,761	2,390	87	1,931	70	914	47
7	February 15, 2021	3,613	2,854	79	2,265	63	1,489	66
Total		68,169	35,684	52	28,383	42	4,634	16

Notes: This table provides weekly summary statistics on all RT-PCR SARS-CoV-2 positive cases from December 21, 2020 to February 21, 2021. Weeks run from Monday to Sunday. It shows the total number of positive cases, the number of case samples selected for WGS, the number that were successfully sequenced, and the number with lineage B.1.1.7. Percentage of B.1.1.7 is calculated out of those with a successfully sequenced genome.

Lineage B.1.1.7 became increasingly dominant, crowding out other lineages, from December 2020 to February 21, 2021, (Figure S1).

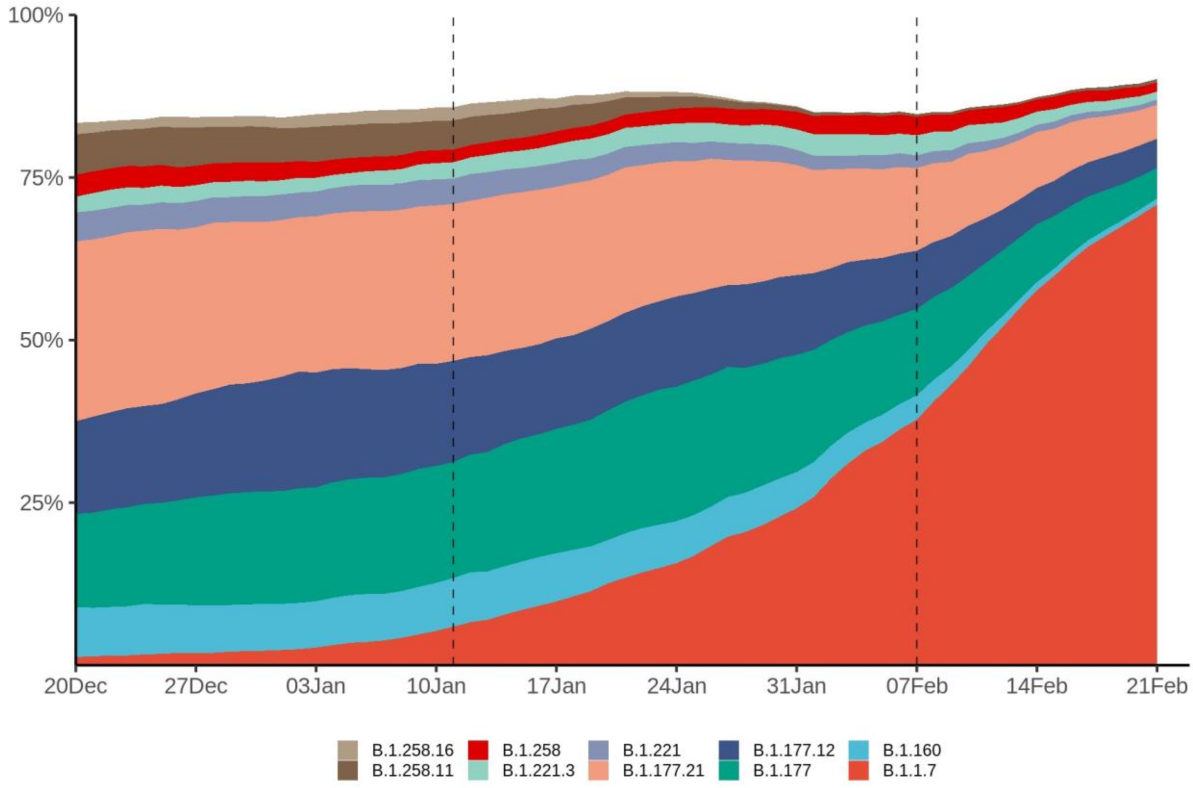

Figure S1: Frequency of detected WGS strains in Denmark over time.

Notes: This figure shows the ten most abundant lineages for cases with a complete genome in Denmark during the study period. Less abundant lineages are included in the white space. 14-day rolling average.

The proportion of cases being sampled varied over time depending on whether the cases occurred in TCDK or in hospitals (Figure S2).

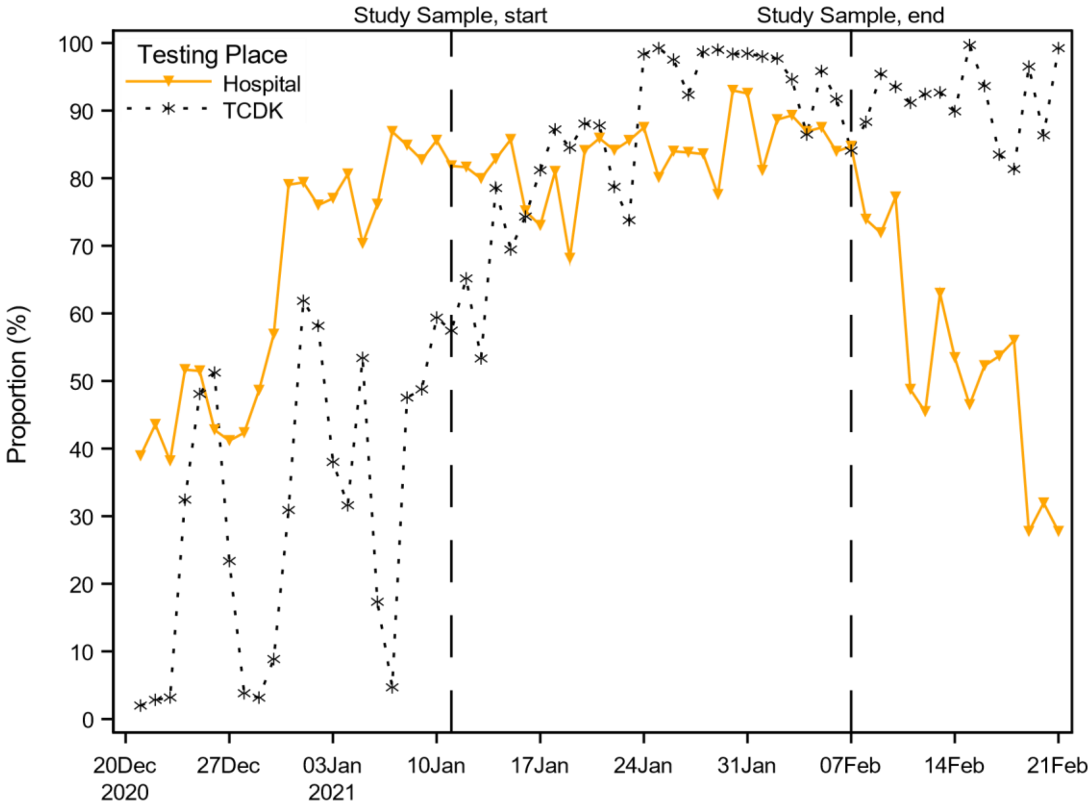

Figure S2: Proportion of positive RT-PCR tests sampled for WGS, stratified by testing facility

Notes: This figure shows the proportion of positive RT-PCR test cases that were selected for WGS stratified by testing facility (TCDK or hospital).

The proportion of cases selected for WGS and the proportion that came back with a genome is dependent on the Ct value (Figure S3). For positive tests with a Ct value of 18, 85% of the samples were selected for WGS (purple) and 76% came back with a genome (green). Thus, the success rate was 89% (76/85). Similarly, for positive tests with a Ct value of 38, 37% of the samples were selected for WGS and 5% came back with a genome. Thus, the success rate was 14% (5/37). The success rate starts to decline for tests with a Ct value ≥30.

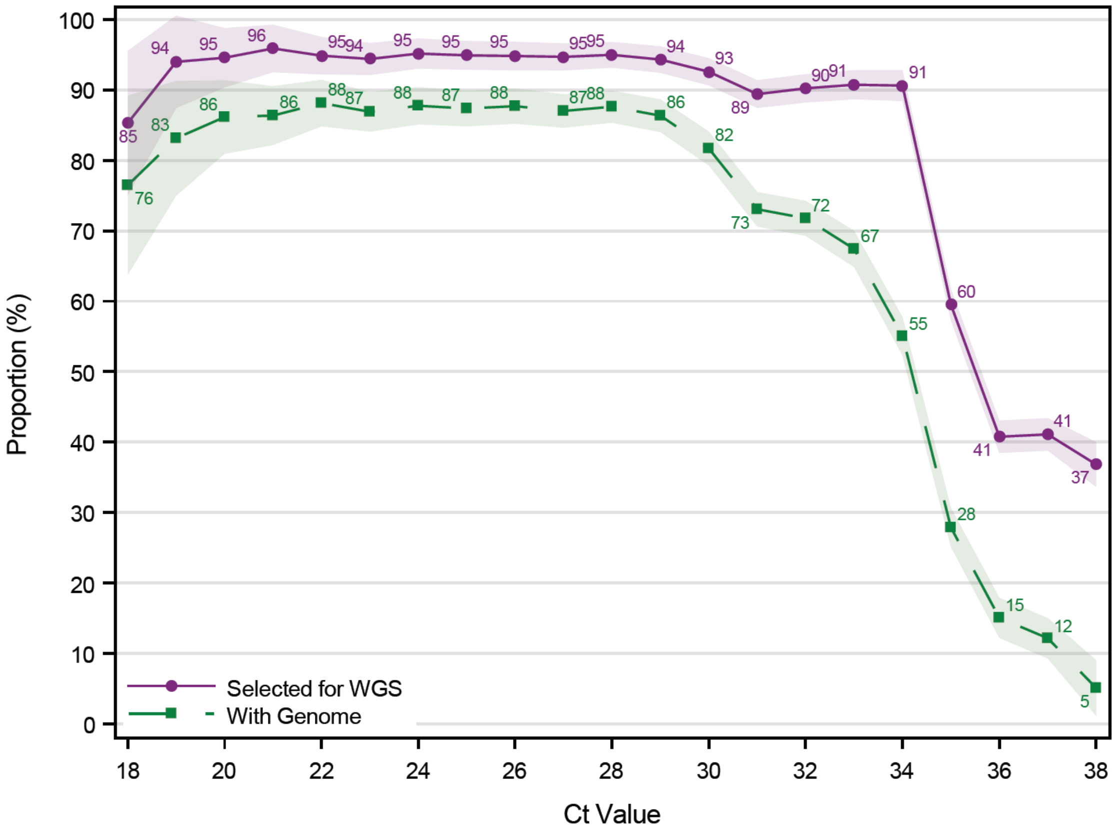

Figure S3: Proportion of positive RT-PCR tests sampled for WGS and with a successfully sequenced genome, by Ct value

Notes: This figure shows the proportion of cases selected for WGS and the proportion that were successfully sequenced stratified by the Ct value of the sample. For positive tests with a Ct value of 18, 85% of the samples were selected for WGS (purple) and 76% came back with a genome (green). Thus, the success rate was 89% (76/85). Similarly, for positive tests with a Ct value of 38, 37% of the samples were selected for WGS and 5% came back with a genome. Thus, the success rate was 14% (5/37). Only samples from TCDK are included and only from the study period (week 2 to 5, 2021). An RT-PCR test is positive if the Ct value is ≤38. The markers show the estimates of the mean. The shaded areas show the 95% confidence bands of the estimates clustered on the household level.

The proportion of cases being sampled for WGS dependent on the Ct value varies over time (Figure S4). In week 2 TCDK started to sample systematically and to sample on Ct values. From Figure S4, we see that in week 2, TCDK used a Ct value cut-off of 30, 32, and 35. In weeks 3-6, TCDK used a Ct value cut-off of 35. Samples with higher Ct values (35<Ct≤38) were included, when WGS capacity allowed for it.

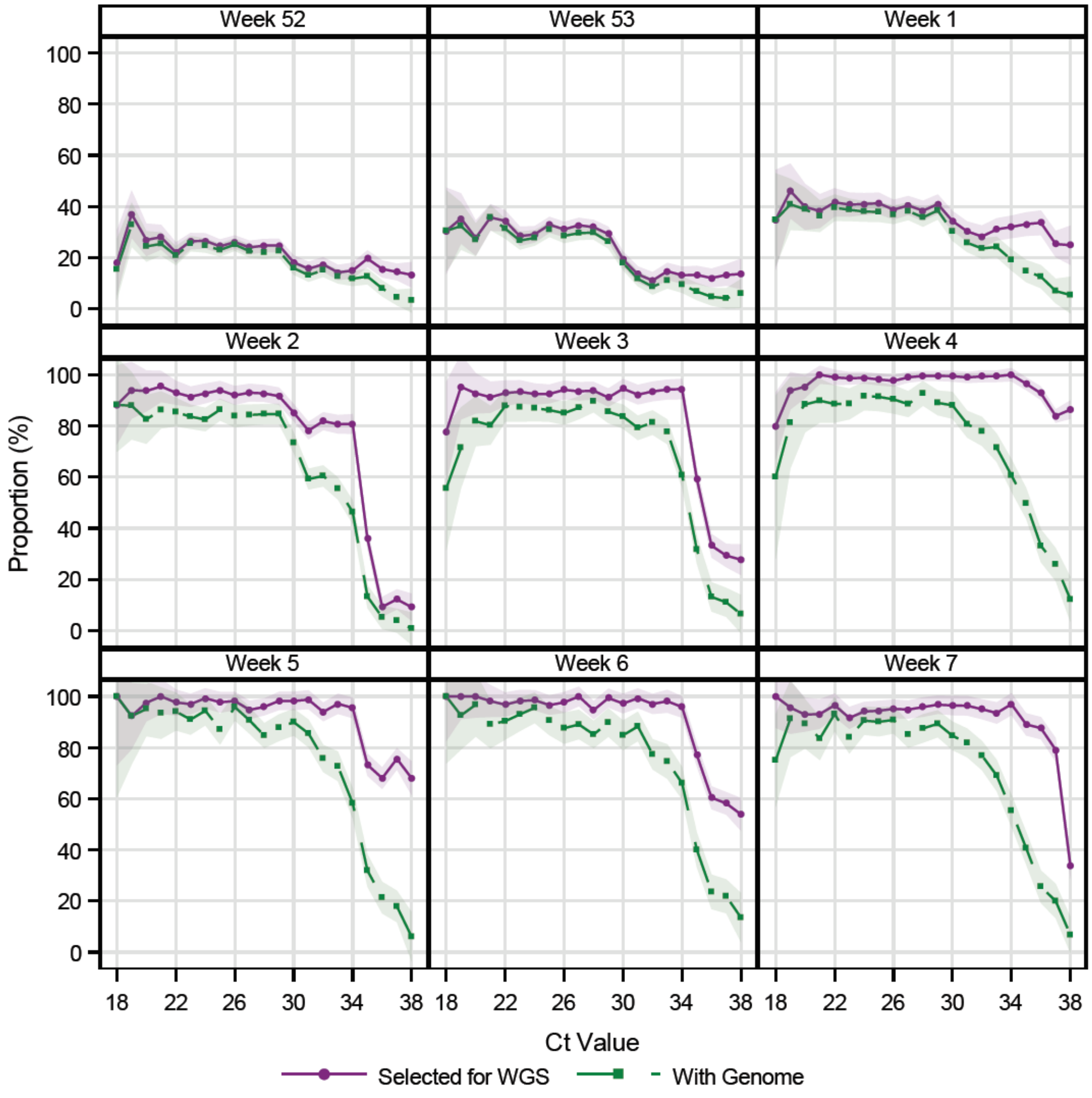

Figure S4: Proportion of positive RT-PCR tests sampled for WGS and with a genome, by Ct value and calendar week

Notes: This figure shows the proportion of cases selected for WGS and the proportion that were successfully sequenced stratified by the Ct value of the sample, across weeks. Only samples from TCDK are included. An RT-PCR test is positive if the Ct value is ≤38. The markers show the estimates of the mean. The shaded areas show the 95% confidence bands of the estimates clustered on the household level.

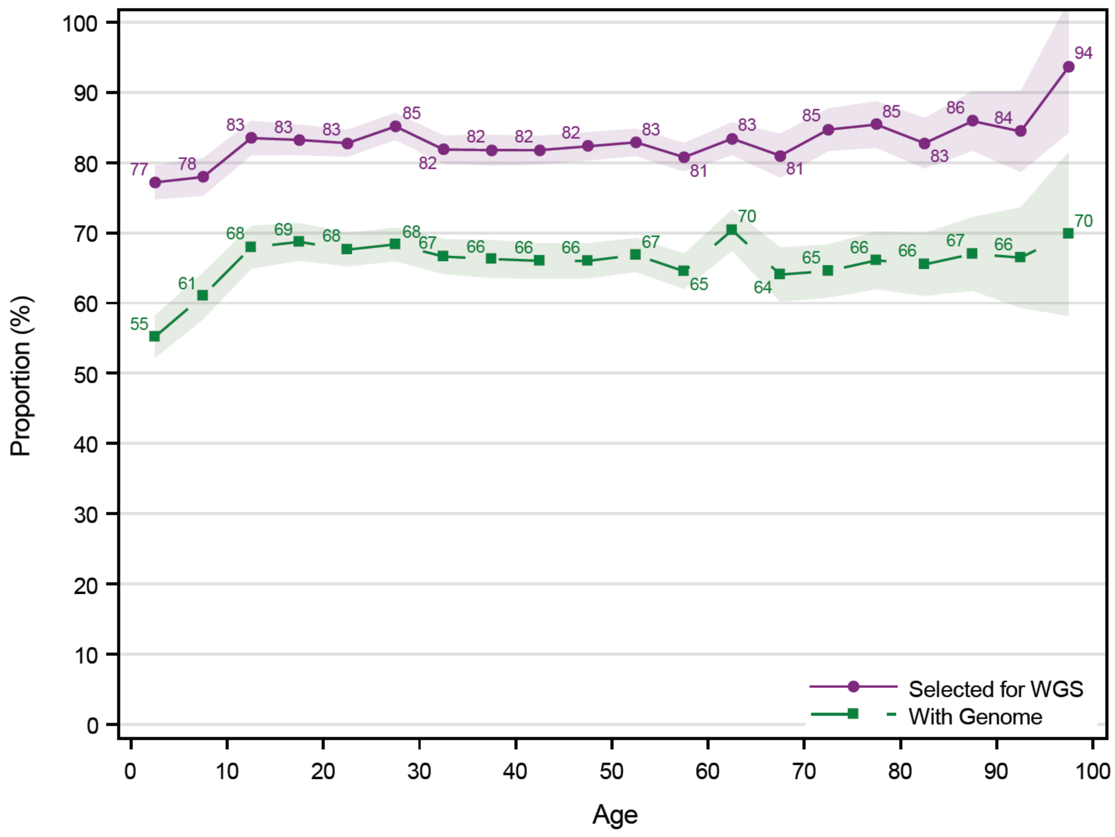

Figure S5: Proportion of positive RT-PCR tests sampled for WGS and with a successfully sequenced genome, by age.

Notes: This figure shows the proportion of cases selected for WGS and the proportion that were successfully sequenced stratified by the age of the samples person. Only samples from the study period (week 2 to 5, 2021) were included. An RT-PCR test is positive if the Ct value is ≤38. The markers show the estimates of the mean. The shaded areas show the 95% confidence bands of the estimates clustered on the household level.

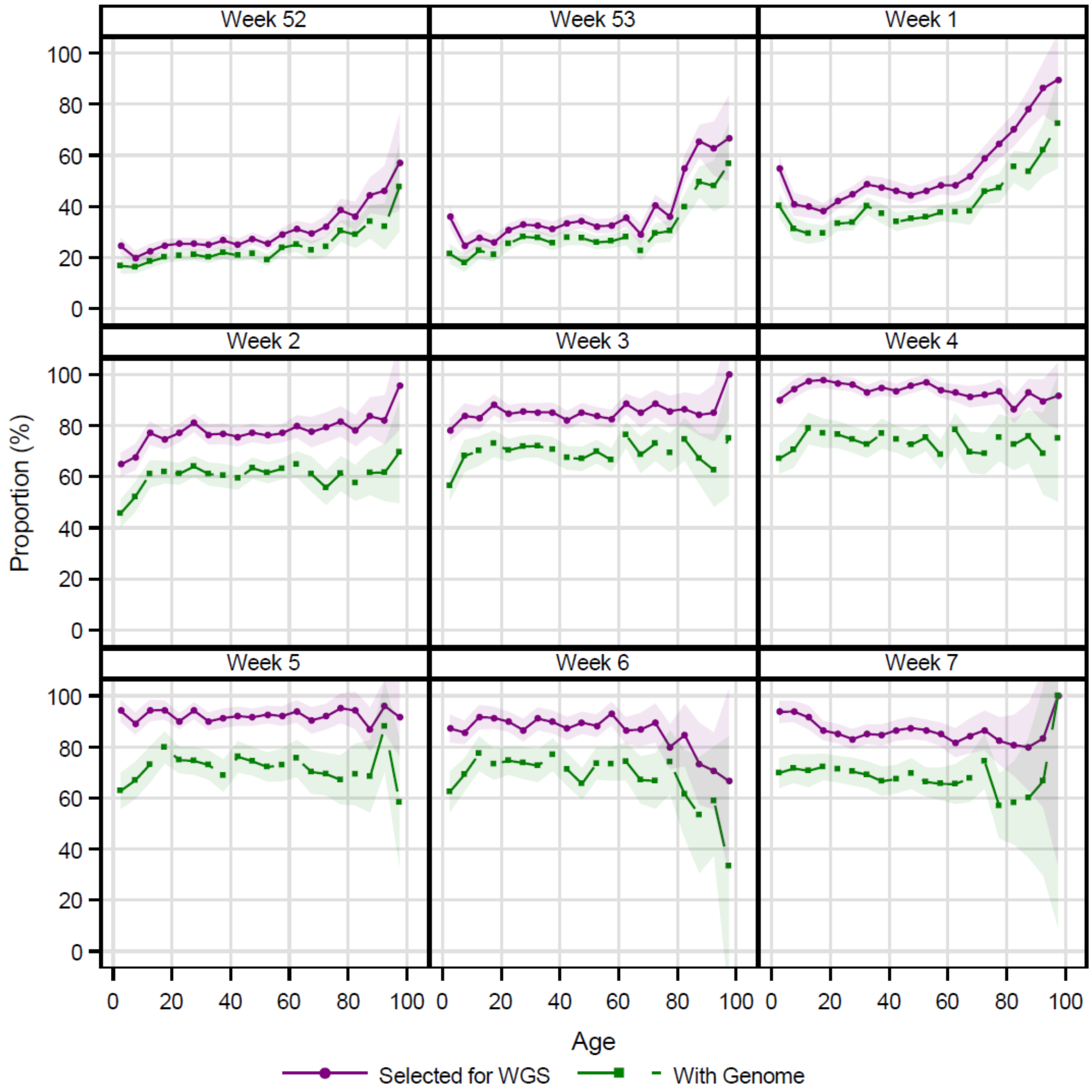

Figure S6: Proportion of positive RT-PCR tests sampled for WGS and with a genome, by age and calendar week.

Notes: This figure shows the proportion of cases selected for WGS and the proportion that were successfully sequenced stratified by the age of the samples person, across weeks. Only samples from TCDK are included. An RT-PCR test is positive if the Ct value is ≤38. The markers show the estimates of the mean. The shaded areas show the 95% confidence bands of the estimates clustered on the household level.

The distribution of Ct values of the cases stratified by B.1.1.7 (red), other lineages (blue) are relatively similar, while samples with no genome (gray) have a distribution with higher Ct values (Figure S7).

WGS was mainly obtained for samples with low Ct values compared with the distribution of Ct values from the whole population (gray dotted line in Figure S7). We found that the Ct value distribution for B.1.1.7 and other lineages were approximately similar from week 1 to week 7 (Figure S7). We see a clear shift in the distribution of cases without a successfully sequenced genome from week 2, when SSI started to systematically select case samples on Ct values.

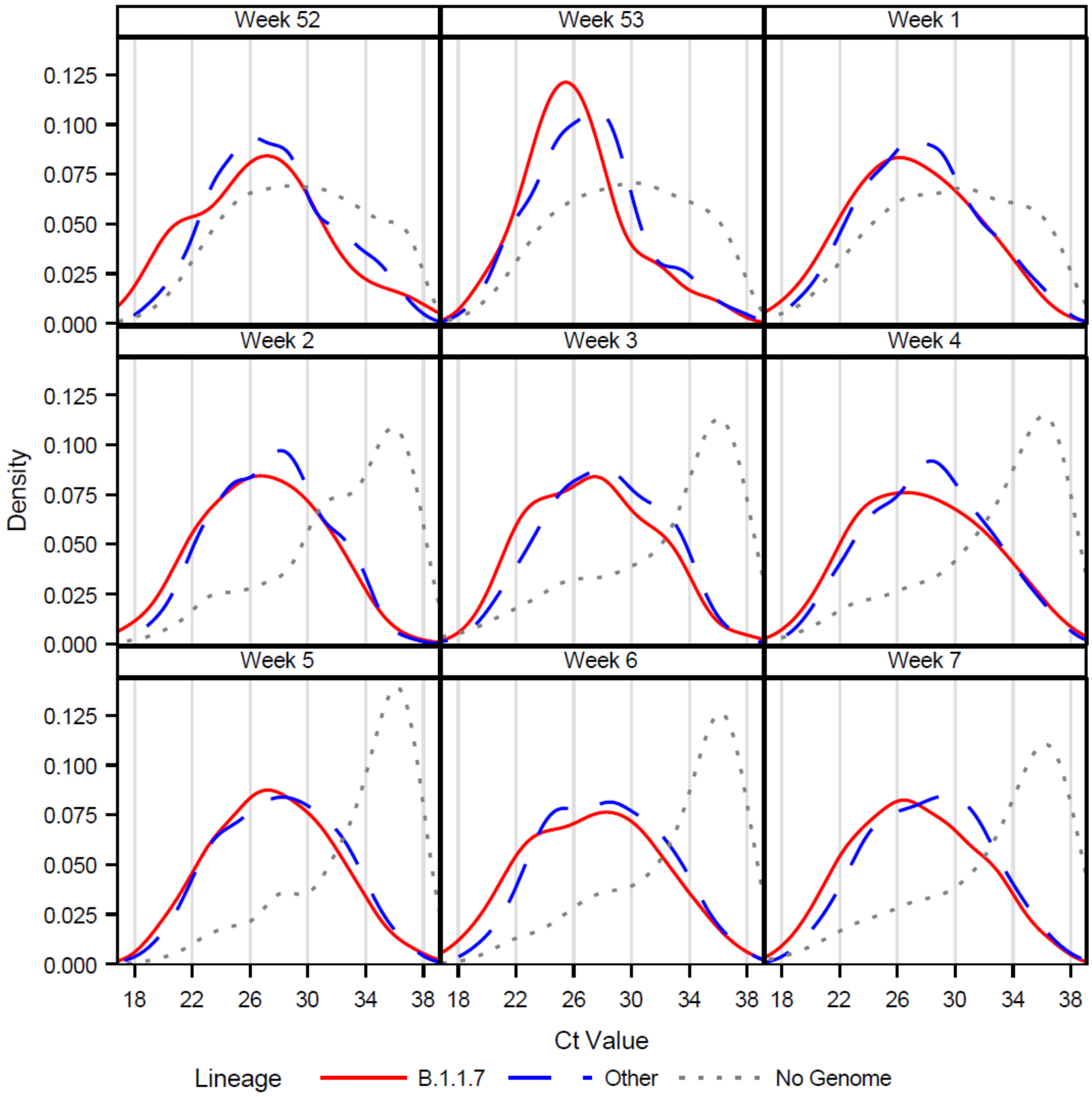

Figure S7: Ct Value distributions by calendar week

Notes: This figure shows the kernel density distributions of the Ct value for cases infected with B.1.1.7 (red), other lineages (blue), and without a successfully sequenced genome (gray). In week 2, 2021, SSI started systematic sampling on Ct values from tests from TCDK. Only samples from TCDK are included. An RT-PCR test is positive if the Ct value is ≤38.

The distribution of the age of the cases stratified by B.1.1.7 (red), other lineages (blue) are relatively similar, although B.1.1.7 seems to mainly infect younger people in weeks 2-4 (Figure S8).

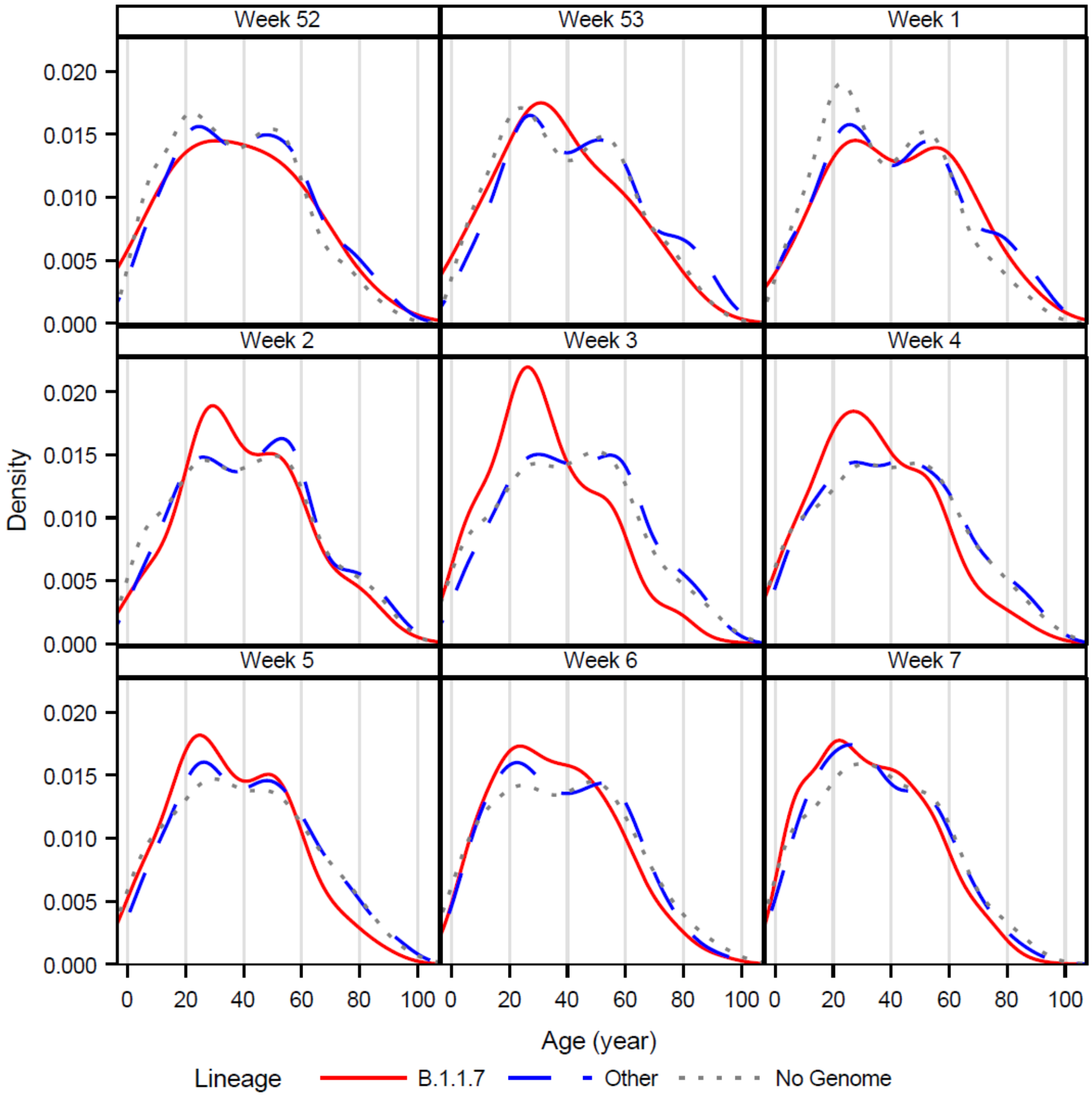

Figure S8: Age distributions by calendar week

Notes: This figure shows kernel density distributions of the age for cases infected with B.1.1.7 (red), other lineages (blue), and without a successfully sequenced genome (gray).

Supplementary Note 3: Additional Analyses

Table S2: Summary statistics

	Primary Cases				Potential	Positive	Attack	
		Selected	With	With	Secondary	Secondary	Rate	
	Total	for WGS	Genome	B.1.1.7	Cases	Cases	(%)	(95%-CI)
**Total**	8,093	6,632	5,241	808	16,612	4,133	25	(24-26)
**Sex**								
Male	3,648	3,013	2,406	419	8,905	2,190	25	(24-26)
Female	4,445	3,619	2,835	389	7,707	1,943	25	(24-26)
**Age**								
0 - 5	274	207	148	36	1,975	410	21	(19-23)
5 - 10	145	120	89	18	1,515	412	27	(25-30)
10 - 15	229	196	159	29	1,684	424	25	(23-27)
15 - 20	566	474	398	62	1,586	331	21	(19-23)
20 - 25	743	620	495	104	1,217	233	19	(17-21)
25 - 30	788	674	525	100	1,130	261	23	(21-26)
30 - 35	732	591	473	83	1,017	263	26	(23-29)
35 - 40	621	510	397	60	859	220	26	(23-29)
40 - 45	734	588	464	61	1,025	245	24	(21-27)
45 - 50	730	594	456	58	1,142	276	24	(22-27)
50 - 55	755	620	495	79	1,147	291	25	(23-28)
55 - 60	688	546	422	53	873	245	28	(25-31)
60 - 65	466	382	324	26	609	203	33	(30-37)
65 - 70	203	157	125	12	310	112	36	(31-42)
70 - 75	173	145	105	12	235	87	37	(31-43)
75 - 80	127	110	85	11	157	63	40	(32-48)
80 - 85	78	64	56	<5	96	45	47	(37-57)
85 - 90	41	34	25	<5	30	12	40	(22-58)
90 - 95	-	-	-	-	<5	0	0	(.)
>95	-	-	-	-	<5	0	0	(.)
**Household Size**								
2	3,308	2,717	2,108	298	3,308	1,019	31	(29-32)
3	1,886	1,552	1,235	189	3,635	843	23	(22-25)
4	1,848	1,488	1,178	193	5,368	1,292	24	(23-26)
5	790	659	534	92	3,042	714	23	(21-26)
6	261	220	186	36	1,259	265	21	(17-25)

Notes: This table provides summary statistics for the number of primary cases, potential secondary cases, positive secondary cases, and attack rates in the study, stratified by sex, age and household sizes. This table is the same as Table 1, except that age is separated into five-year age groups. 95% confidence intervals are clustered on the household level.

Table S3: Summary statistics split by primary case Ct value and their associated secondary cases

Ct Value	Primary Cases	Potential Secondary Cases	Positive Secondary Cases	Attack Rate (%)	(95%-CI)
**B.1.1.7**					
<18	5	12	9	75	(46-104)
18-20	26	57	28	49	(36-63)
20-22	56	134	58	43	(31-56)
22-24	98	198	78	39	(31-48)
24-26	107	225	76	34	(26-41)
26-28	115	236	84	36	(27-44)
28-30	119	245	97	40	(32-47)
30-32	75	158	58	37	(26-47)
32-34	52	121	41	34	(21-46)
34-36	15	35	11	31	(11-51)
36-38	12	24	8	33	(6-61)
Total With Ct	680	1,445	548	38	(35-41)
Without Ct	128	274	108	39	(31-48)
Total	808	1,719	656	38	(35-41)
**Other Lineages**					
<18	6	14	8	57	(29-86)
18-20	76	168	83	49	(39-59)
20-22	225	472	159	34	(28-40)
22-24	448	923	296	32	(28-36)
24-26	550	1,124	291	26	(23-29)
26-28	658	1,332	359	27	(24-30)
28-30	588	1,244	315	25	(22-28)
30-32	470	1,015	239	24	(20-27)
32-34	326	726	172	24	(20-28)
34-36	109	227	51	22	(16-29)
36-38	36	72	9	13	(2-23)
Total With Ct	3,492	7,317	1,982	27	(26-28)
Without Ct	941	1,798	507	28	(26-31)
Total	4,433	9,115	2,489	27	(26-28)
**No Genome**					
<18	15	30	<5	7	(-6-21)
18-20	26	51	16	31	(17-46)
20-22	53	107	26	24	(13-36)
22-24	101	180	59	33	(25-41)
24-26	99	216	54	25	(17-33)
26-28	115	234	63	27	(19-35)
28-30	155	313	59	19	(14-24)
30-32	234	498	112	22	(18-27)
32-34	308	666	106	16	(12-19)
34-36	491	979	166	17	(14-20)
36-38	476	1,011	99	10	(7-12)
Total With Ct	2,073	4,285	761	18	(16-19)
Without Ct	779	1,493	227	15	(13-18)
Total	2,852	5,778	988	17	(16-18)

Notes: This table provides summary statistics for the number of primary cases divided by their sample Ct value together with their associated potential secondary cases and positive secondary cases. 95% confidence intervals are clustered on the household level.

Table S4 Summary statistics split by primary case age and their associated secondary cases

Age	Primary Cases	Potential Secondary Cases	Positive Secondary Cases	(%)	Attack Rate (95%-CI)
**B.1.1.7**					
0-10	54	141	64	45	(34-57)
10-20	91	242	60	25	(17-33)
20-30	204	380	104	27	(21-34)
30-40	143	343	134	39	(32-46)
40-50	119	293	124	42	(35-50)
50-60	132	234	120	51	(43-59)
60-70	38	50	31	62	(49-75)
70-80	23	32	16	50	(32-68)
80-90	<5	<5	<5	75	(33-117)
Total	808	1,719	656	38	(35-41)
**Other Lineages**					
0-10	183	467	113	15	(11-20)
10-20	466	1,206	202	9	(6-13)
20-30	816	1,495	299	12	(10-15)
30-40	727	1,868	520	19	(16-22)
40-50	801	1,991	596	19	(16-22)
50-60	785	1,303	432	17	(14-20)
60-70	411	508	201	25	(19-30)
70-80	167	197	83	32	(24-41)
80-90	77	80	43	28	(13-42)
Total	4,433	9,115	2,489	27	(26-28)
**No Genome**					
0-10	182	472	73	24	(19-29)
10-20	238	594	56	17	(14-20)
20-30	511	930	116	20	(18-22)
30-40	483	1,191	228	28	(25-31)
40-50	544	1,323	257	30	(27-33)
50-60	526	846	144	33	(30-36)
60-70	220	268	66	40	(35-44)
70-80	110	114	37	42	(35-50)
80-90	38	40	11	54	(42-65)
Total	2,852	5,778	988	17	(16-18)

Notes: This table provides summary statistics for the number of primary cases divided by their age together with their associated potential secondary cases and positive secondary cases. 95% confidence intervals are clustered on the household level.

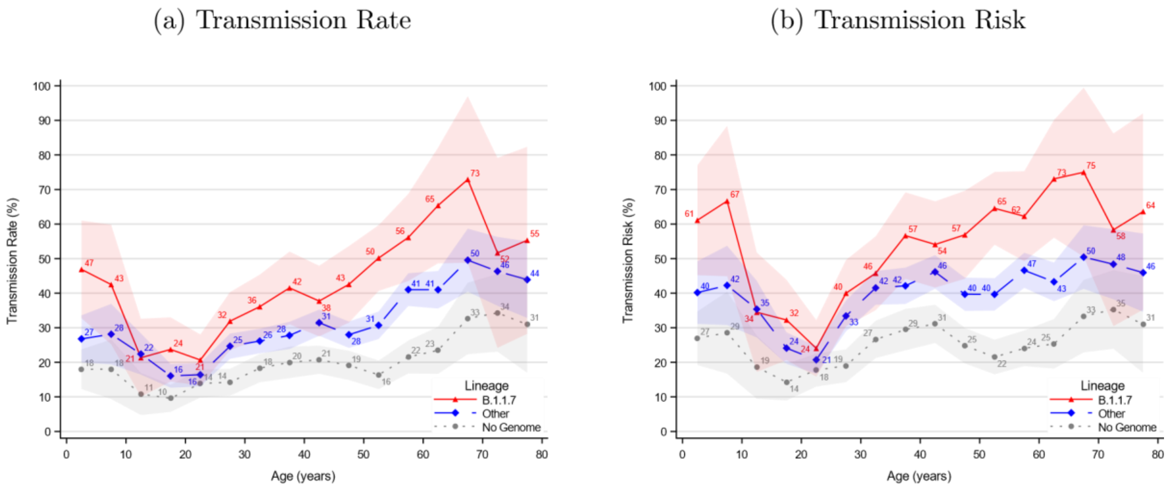

Figure S9: Age structured transmissibility stratified by lineage in five-year age groups.

Notes: The transmission rate describes the proportion of potential secondary cases within the household that were infected. The transmission risk describes the proportion of infected primary cases that infected at least one secondary case. This figure is the same as Figure 1, except that it shows five-year age groups.

The markers show the estimates of the mean. The shaded areas show the 95% confidence bands of the estimates clustered on the household level.

Primary cases infected with B.1.1.7 generally had a higher transmissibility compared with cases infected with other lineages, across Ct values (Figure S8 and S9).

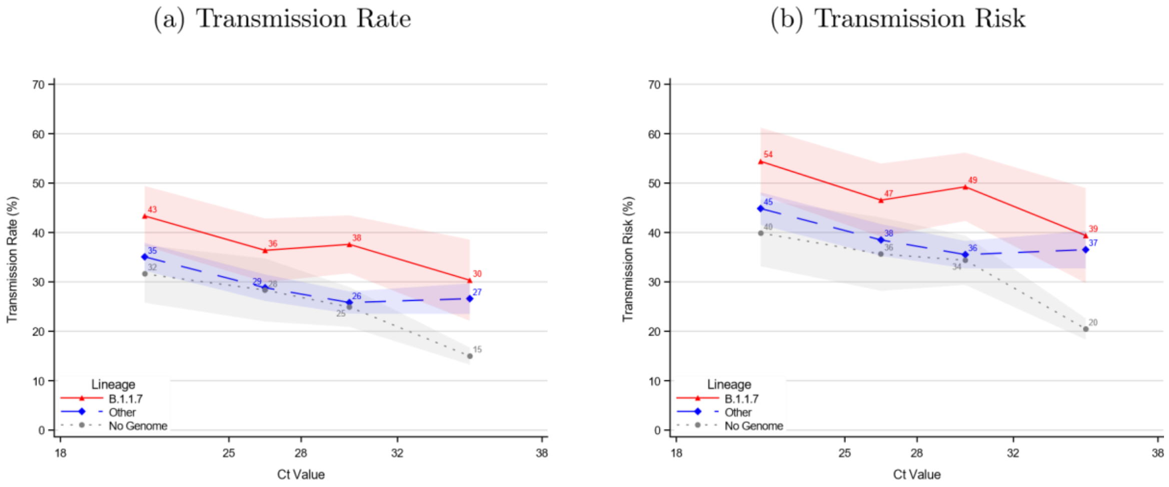

Figure S10: Transmissibility stratified by lineage and Ct value quartiles

Notes: The transmission rate describes the proportion of potential secondary cases within the household that were infected. The transmission risk describes the proportion of infected primary cases that infected at least one secondary case. The markers show the estimates of the mean. The shaded areas show the 95% confidence bands of the estimates clustered on the household level.

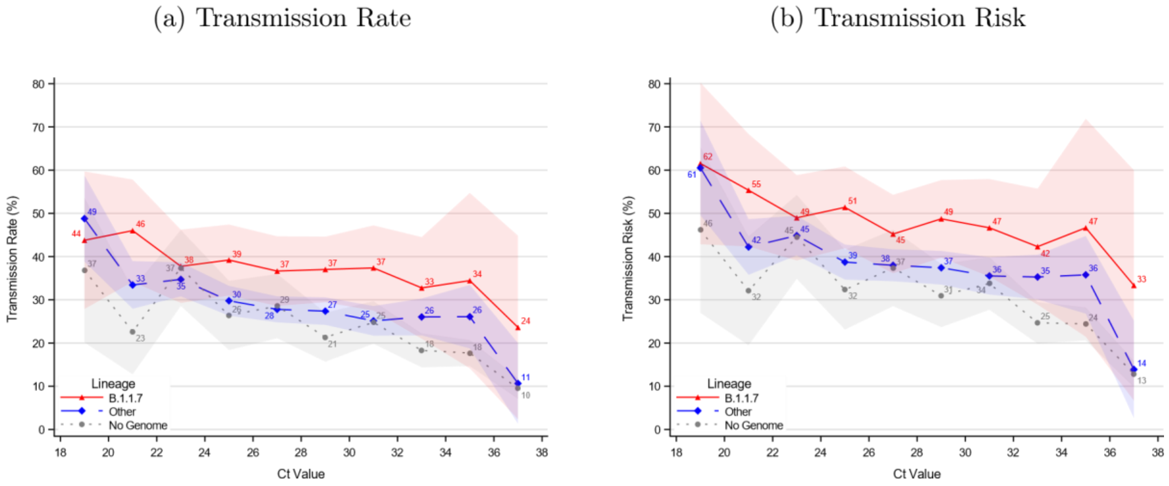

Figure S11: Transmissibility stratified by lineage and Ct values

Notes: The transmission rate describes the proportion of potential secondary cases within the household that were infected. The transmission risk describes the proportion of infected primary cases that infected at least one secondary case. The markers show the estimates of the mean. The shaded areas show the 95% confidence bands of the estimates clustered on the household level.

Table S5: Sensitivity analysis for the definition of co-primary cases: Odds ratio estimates

	I	II	III	IV
Days for including Sec. Cases	1-14	2-14	3-14	4-14
Transmission Rate, B.1.1.7	1.62	1.61	1.59	1.60
95%-CI	(1.39-1.90)	(1.37-1.89)	(1.35-1.88)	(1.34-1.90)
Transmission Risk, B.1.1.7	1.61	1.65	1.67	1.65
95%-CI	(1.36-1.90)	(1.38-1.98)	(1.37-2.03)	(1.34-2.04)
Constant	✓	✓	✓	✓
Age, Primary Case	✓	✓	✓	✓
Ct Value	✓	✓	✓	✓
Observations	8,762	8,590	8,348	8,224
Households	4,172	4,033	3,847	3,761

Notes: This table shows sensitivity analysis results for the transmission rate and transmission risk when restricting the inclusion criteria for secondary cases. Column I includes secondary cases that tested positive on days 1-14, i.e., the same as in the paper. Column II only includes secondary cases that tested positive on days 2-14. Column III includes secondary cases that tested positive on days 3-14. Column IV includes secondary cases that tested positive on days 4-14. 95% confidence intervals clustered on the household level.

Table S6: Odds ratio estimates for transmissibility for B.1.1.7 compared with other lineages, excluding households with co-primary cases

	**Transmission Rate**				**Transmission Risk**		
	**I**	**II**	**III**	**IV**	**V**	**VI**	**VII**
B.1.1.7	1.49	1.70	1.70	1.64	1.52	1.68	1.63
95%-CI	(1.29-1.72)	(1.46-1.97)	(1.47-1.97)	(1.39-1.93)	(1.30-1.78)	(1.43-1.97)	(1.37-1.94)
Constant	✓	✓	✓	✓	✓	✓	✓
Age, Primary Case		✓	✓	✓		✓	✓
Age, Pot. Sec. Case			✓	✓			
Ct Value				✓			✓
Observations	10,295	10,295	10,295	8,286	10,295	10,295	8,286
Households	4,948	4,948	4,948	3,912	4,948	4,948	3,912

Notes: This table is comparable to Table 3, but excluding households with co-primary cases, i.e., more than one (primary) case identified on day zero. Columns I-IV provide odds ratio estimates for the increased transmission rate of B.1.1.7 compared with other lineages. Columns V-VII show the same for the transmission risk. Column I provides the crude estimates, i.e., only with a constant and without any controls. Column II further includes fixed effects for ten-year age groups of the primary cases. Column III further includes the age of potential secondary cases. Column IV further includes fixed effects for Ct values in bi-value groups. This sample is further restricted to only include primary cases identified in TCDK, as we only have Ct values on those. Column V provides the crude estimates, i.e., only with a constant and without any controls. Column VI further includes fixed effects for ten-year age groups of the primary cases. Column VII further includes fixed effects for Ct values in bi-value groups. This sample is further restricted to only include primary cases identified in TCDK, as we only have Ct values on those. All effects are included as fixed effects. Pot. Sec. Case = Potential Secondary Cases. Only primary cases identified in TCDK are included in models with Ct values. 95% confidence intervals clustered on the household level.

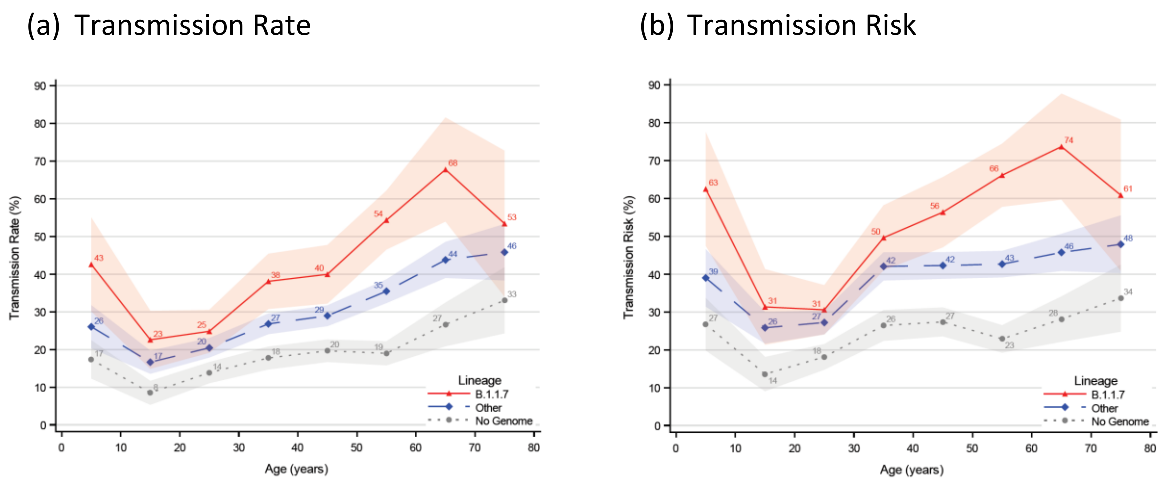

Figure S12: Age structured transmissibility stratified by lineage of the primary case, , excluding households with co-primary cases

Notes: This figure is comparable to Figure 1, but excluding households with co-primary cases, i.e., more than one (primary) case identified on day zero. The transmission rate describes the proportion of potential secondary cases within the household that were infected. The transmission risk describes the proportion of infected primary cases that infected at least one secondary case. Figure S7 provides the same graphs for five-year age groups. The markers show the estimates of the mean. The shaded areas show the 95% confidence bands of the estimates clustered on the household level.

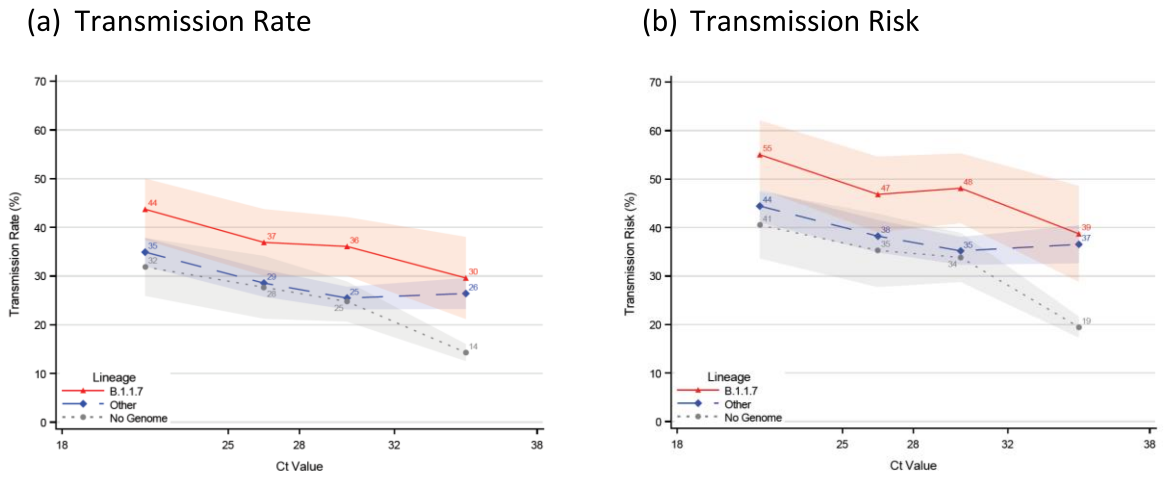

Figure S13: Transmissibility stratified by lineage and Ct value quartiles, excluding households with co-primary cases

Notes: This figure is comparable to Figure S8, but excluding households with co-primary cases, i.e., more than one (primary) case identified on day zero. The transmission rate describes the proportion of potential secondary cases within the household that were infected. The transmission risk describes the proportion of infected primary cases that infected at least one secondary case. The markers show the estimates of the mean. The shaded areas show the 95% confidence bands of the estimates clustered on the household level.

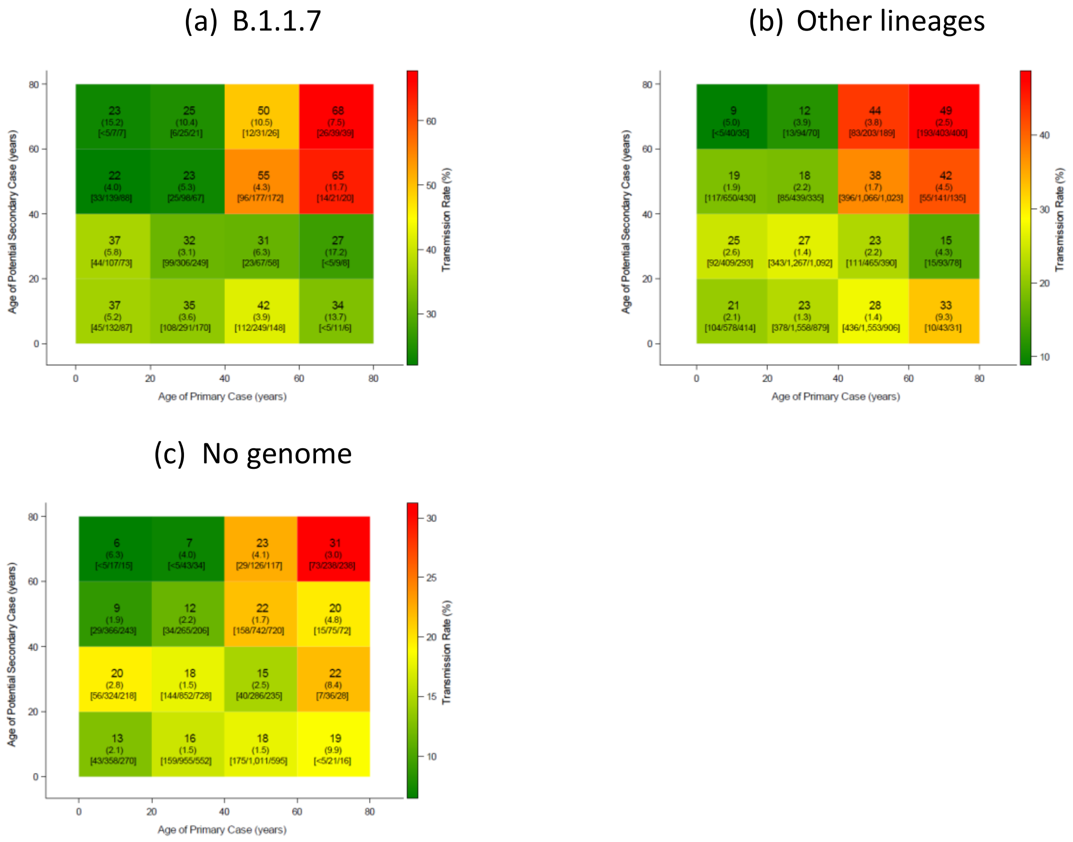

Figure S14: Age-by-age transmission rate stratified by lineage

Notes: This figure is provides estimates of the transmission rate for each combination of age of the primary and potential secondary case, stratified by the lineage of the primary case. (.) Standard errors of the estimates are clustered on the household level. [./././] Number of primary cases / number of potential secondary cases / number of positive secondary cases.

Supplementary Note 3: Statistical Analyses

Age Structured Transmissibility

To estimate the association between age and transmission rate, stratified by lineage, we estimated the non-parametric regression equation:

$$y_p = \beta \times Age_{p,10} + \varepsilon_p \, , \tag{1}$$

where $Age_{p,10}$ is the age (in ten-year groups) of the primary case. β measures the transmission rate for each ten-year age group of the primary cases. ε_p denotes the error term, clustered on the household (event) level.

Additive vs. Multiplicative Effect of B.1.1.7 Transmissibility

We wanted to evaluate whether the effect of being infected with B.1.1.7 relative to being infected with other lineages was additive or multiplicative, which is important for designing proper simulation models. With binomial outcomes the canonical link function is the logit function which corresponds to a multiplicative effect. An additive effect of the covariates can be modelled by using the identity link in a generalized linear regression model.

Thus, to estimate the transmissibility effect of B.1.1.7 compared with other lineages, we estimated the model with the following linear predictor:

$$\eta = Constant + B.1.1.7 + Age_p + Age_{p,s} + Ct_p + \varepsilon_p \, , \tag{2}$$

while varying the link function to compare the model fit of an additive versus a multiplicative effect.

As the two models include the same parameters, the model fits can be compared using the Akaike Information Criterion (AIC). Furthermore, reduced versions of the linear predictors were tested. Across all three model specifications and for both transmission rate and transmission risk, we found that the logit model had a lower AIC and, thereby, was a better fit

compared with the identity model, implying that the increased transmissibility is multiplicative and not additive (Table S7 and S8).

Table S7: Comparison of additive vs. multiplicative effect, Transmission Rate

	Linear	Logit	Linear	Logit	Linear	Logit
AIC	6,280	6,277	6,302	6,273	4,963	4,953
B.1.1.7	✓	✓	✓	✓	✓	✓
Constant	✓	✓	✓	✓	✓	✓
Age, Primary Case	✓	✓	✓	✓	✓	✓
Age, Pot. Sec. Case			✓	✓	✓	✓
Ct Value					✓	✓
Observations	10,834	10,834	10,834	10,834	8,762	8,762
Households	5,241	5,241	5,241	5,241	4,172	4,172

Notes: This table provides a comparison of an additive and multiplicative model using the Akaike Information Criteria (AIC). The transmission rate describes the proportion of potential secondary cases within the household that were infected.

Table S8: Comparison of additive vs. multiplicative effect, Transmission Risk

	Linear	Logit	Linear	Logit
AIC	6,900	6,898	5,455	5,455
B.1.1.7	✓	✓	✓	✓
Constant	✓	✓	✓	✓
Age, Primary Case	✓	✓	✓	✓
Ct Value			✓	✓
Observations	10,834	10,834	8,762	8,762
Households	5,241	5,241	4,172	4,172

Notes: This table provides a comparison of an additive and multiplicative model using the Akaike Information Criteria (AIC). The transmission risk describes the proportion of infected primary cases that infected at least one secondary case.
