# Supplementary material for: Increased transmissibility of SARS-CoV-2 lineage B.1.1.7 by age and viral load
Source: Nat Commun. 2021 Dec 13;12:7251. doi: 10.1038/s41467-021-27202-x (PMC8669007; doi:10.1038/s41467-021-27202-x)
Supplement: Supplementary file 3 — Reporting Summary [file 41467_2021_27202_MOESM3_ESM.pdf]

nature research

Corresponding author(s): Frederik Plesner Lyngse

Last updated by author(s): Oct 20, 2021

Reporting Summary

Nature Research wishes to improve the reproducibility of the work that we publish. This form provides structure for consistency and transparency in reporting. For further information on Nature Research policies, see our Editorial Policies and the Editorial Policy Checklist.

Statistics

For all statistical analyses, confirm that the following items are present in the figure legend, table legend, main text, or Methods section.

n/a	Confirmed	
☐	☒	The exact sample size (*n*) for each experimental group/condition, given as a discrete number and unit of measurement
☐	☒	A statement on whether measurements were taken from distinct samples or whether the same sample was measured repeatedly
☒	☐	The statistical test(s) used AND whether they are one- or two-sided<br>*Only common tests should be described solely by name; describe more complex techniques in the Methods section.*
☐	☒	A description of all covariates tested
☐	☒	A description of any assumptions or corrections, such as tests of normality and adjustment for multiple comparisons
☐	☒	A full description of the statistical parameters including central tendency (e.g. means) or other basic estimates (e.g. regression coefficient) AND variation (e.g. standard deviation) or associated estimates of uncertainty (e.g. confidence intervals)
☐	☒	For null hypothesis testing, the test statistic (e.g. *F*, *t*, *r*) with confidence intervals, effect sizes, degrees of freedom and *P* value noted<br>*Give P values as exact values whenever suitable.*
☒	☐	For Bayesian analysis, information on the choice of priors and Markov chain Monte Carlo settings
☒	☐	For hierarchical and complex designs, identification of the appropriate level for tests and full reporting of outcomes
☒	☐	Estimates of effect sizes (e.g. Cohen's *d*, Pearson's *r*), indicating how they were calculated

Our web collection on statistics for biologists contains articles on many of the points above.

Software and code

Policy information about availability of computer code

Data collection	We performed no data collection and performed no genome sequencing for this study.
Data analysis	We used SAS 9.4 to manage and analyze the data. The code used for this study is deposited at GitHub: https://github.com/Flyngse/SARS-CoV-2_B.1.1.7_Transmissibility.git.

For manuscripts utilizing custom algorithms or software that are central to the research but not yet described in published literature, software must be made available to editors and reviewers. We strongly encourage code deposition in a community repository (e.g. GitHub). See the Nature Research guidelines for submitting code & software for further information.

Data

Policy information about availability of data

All manuscripts must include a data availability statement. This statement should provide the following information, where applicable:

- Accession codes, unique identifiers, or web links for publicly available datasets
- A list of figures that have associated raw data
- A description of any restrictions on data availability

The data used in this study are available under restricted access due to Danish data protec-tion legislation. The data are available for research upon reasonable request to The Danish Health Data Authority and Statens Serum Institut and within the framework of the Danish data protection legislation and any required permission from Authorities. We performed no data collection and performed no sequencing for this study. The Danish public health author-ities deposits all SARS-CoV-2 sequences to GISAID (https://www.gisaid.org/), including those used in this study. The GISAID accession identifiers are listed in Supplementary File S1.

Field-specific reporting

Please select the one below that is the best fit for your research. If you are not sure, read the appropriate sections before making your selection.

- [x] Life sciences
- [ ] Behavioural & social sciences
- [ ] Ecological, evolutionary & environmental sciences

For a reference copy of the document with all sections, see nature.com/documents/nr-reporting-summary-flat.pdf

Life sciences study design

All studies must disclose on these points even when the disclosure is negative.

Sample size	Study data: All cases with a positive SARS-CoV-2 RT-PCR test during the study period January 11 (week 2) to February 7, 2021 (week 5) and their household members. There were no changes in public health measures or COVID-19 related restrictions in this period, and the period did not include any public holidays. In Appendix A, we provide summary statistics to substantiate our choice of study period. Appendix A, descriptive statistics: All cases with a positive SARS-CoV-2 RT-PCR test from December 20, 2020 (week 52) to February 21, 2021 (week 7).
Data exclusions	We exclude households with less than one and more than six members.
Replication	The code used for this study can be obtained from the corresponding author upon request. The analysis is performed on administrative register data. The analysis can be replicated with data access through the Statens Serum Institut and Danish Health Data Authority.
Randomization	Randomization was not applicable, as the study uses observational data from administrative registers.
Blinding	Blinding was not applicable, as the study uses observational data from administrative registers.

Reporting for specific materials, systems and methods

We require information from authors about some types of materials, experimental systems and methods used in many studies. Here, indicate whether each material, system or method listed is relevant to your study. If you are not sure if a list item applies to your research, read the appropriate section before selecting a response.

Materials & experimental systems

n/a	Involved in the study	
☒	☐	Antibodies
☒	☐	Eukaryotic cell lines
☒	☐	Palaeontology and archaeology
☒	☐	Animals and other organisms
☒	☐	Human research participants
☒	☐	Clinical data
☒	☐	Dual use research of concern

Methods

n/a	Involved in the study	
☒	☐	ChIP-seq
☒	☐	Flow cytometry
☒	☐	MRI-based neuroimaging
