# Supplementary material for: Increased transmissibility of SARS-CoV-2 lineage B.1.1.7 by age and viral load
Source: Nat Commun. 2021 Dec 13;12:7251. doi: 10.1038/s41467-021-27202-x (PMC8669007; doi:10.1038/s41467-021-27202-x)
Supplement: Supplementary file 4 — Description of Additional Supplementary Files [file 41467_2021_27202_MOESM4_ESM.pdf]

Description of Additional Supplementary Files

Supplementary Data 1: GISAID Accession IDs
